# Effectiveness of radiotherapy or chemoradiotherapy in older patients with advanced-stage, non-surgically treated head and neck cancer: A machine learning analysis

**DOI:** 10.1007/s12032-026-03420-5

**Published:** 2026-10-08

**Authors:** Rasheed Omobolaji Alabi, Alhadi Almangush, Mohammed Elmusrati, Ylva Tiblom Ehrsson, Göran Laurell, Antti A. Mäkitie

**Affiliations:** 1 Research Program in Systems Oncology, Faculty of Medicine, University of Helsinki, Helsinki, Finland; 2 Cyber-Physical Systems (CPS) Research Team, School of Technology and Innovations, University of Vaasa, Vaasa, Finland; 3 Institute of Biomedicine, Pathology, University of Turku, Turku, Finland; 4 Department of Pathology, University of Helsinki, Helsinki, Finland; 5 Libyan Authority for Scientific Research, Tripoli, Libya; 6 Department of Surgical Sciences, Section of Otorhinolaryngology and Head and Neck Surgery, Uppsala University, Uppsala, Sweden; 7 Department of Otorhinolaryngology – Head and Neck Surgery, University of Helsinki, Helsinki University Hospital, Helsinki, Finland; 8 Division of Ear, Nose and Throat Diseases, Department of Clinical Sciences, Intervention and Technology, Karolinska Institute, Karolinska University Hospital, Stockholm, Sweden

**Keywords:** Machine learning, Head and Neck Cancer Squamous Cell Carcinoma (HNSCC), Overall survival, Propensity Score Matching, Chemoradiotherapy

## Abstract

The rising incidence of head and neck squamous cell carcinoma (HNSCC) is linked to both the HPV epidemic and the aging population. As a result, there is an emerging need to develop management strategies for older patients with advanced-stage HNSCC. This study aims to apply machine learning (ML) techniques to compare the effectiveness of definitive concurrent chemoradiotherapy (CRT) and radiotherapy (RT) alone in older patients (≥ 65 years) with stage III-IV non-surgically treated HNSCC. We used Kaplan-Meier (KM) survival curves, Cox proportional hazards models, and ML-based permutation feature importance (PFI) to compare the effectiveness of CRT versus single modality radiotherapy (RT) for disease-specific survival (DSS) in this population of patients with HNSCC. Finally, we used propensity-score matched (PSM) analysis to clarify if there is a significant difference between patients that received either CRT or RT alone. A total of 1525 patients with HNSCC were obtained from the Surveillance, Epidemiology, and End Results (SEER) Program to build the ML model. Furthermore, the PFI analysis evaluated the effectiveness of CRT or RT alone regarding DSS of older (≥ 65 year) patients with HNSCC. Then, we performed a 1:1 propensity-score matched (PSM) analysis to reduce treatment selection bias and to analyze the prognostic role of CRT. Our ML model showed performance accuracy of 55.5% in predicting DSS in this group with HNSCC. The aggregate feature importance showed that treatment option (CRT or RT alone) is among the four most important features for enhancing DSS among these patients. Specifically, our KM curves, Cox proportional hazards model, and PFI analyses all showed that CRT seems more feasible and effective than RT alone for older patients with stage III-IV HNSCC who did not receive surgery. In addition, patients with stage III disease had better survival rates than those with stage IV. The PSM confirmed that there are substantial differences in nearly all covariates between patients who received CRT or RT alone. The results from the SEER database provide insight into a potential survival benefit for CRT in older patients with stage III-IV HNSCC who are not candidates for surgery. This further emphasizes that treatment decisions at an advanced stage may be made according to other patient-related factors rather than chronological (calendar) age alone.

**Supplementary Information:**

The online version contains supplementary material available at https://doi.org/10.1007/s12032-026-03420-5.

## Introduction

Head and neck cancer (HNC) represents one of the ten most common malignancies worldwide, with an estimated 54,000 new cases and about 11,230 deaths in 2022 in the United States [1]. Studies report that 25–40% of patients with HNC are diagnosed at 70 years or older [2–4]. Remarkably, most of these patients typically present at the advanced stage [5]. Therefore, there is an emerging need to develop age-adapted treatment strategies for older and frail patients with advanced head and neck squamous cell carcinoma (HNSCC) [6]. Due to possible treatment-related morbidity and mortality in older patients with HNSCC [7], complex evaluation and pretreatment management are essential [7]. However, there have been growing concerns as to whether older patients in general can benefit from aggressive treatment modalities [7]. Hence, the selection of appropriate treatment for older patients is subject to debate [1]. For example, surgery, which is a mainstay treatment for HNC, has to be carefully considered for older patients with advanced-stage HNC. This is evident considering the high risk of postoperative complications, distant metastasis, and recurrences that is peculiar to advanced stage HNC [8–10].

HNSCC encompasses several sites in the upper aerodigestive tract, and treatment planning involves a multidisciplinary approach to recommend the optimal treatment for each patient. Treatment can be given either as single modality treatment or combined modality treatment, and may include surgery, radiotherapy, chemotherapy, and immunotherapy. The guidelines of several notable societies, such as the National Comprehensive Cancer Network (NCCN), the American Society of Clinical Oncology (ASCO), and the European Society of Medical Oncology (ESMO), have recommended concurrent chemoradiotherapy (CRT) regimen therapy for advanced stage HNC, especially stage IV HNC [11–15]. Owing to the dearth of evidence supporting CRT for older patients with advanced-stage disease [1], there is a great need to examine the efficacy of CRT in older patients with non-surgically treated stage III-IV HNC.

This study aims to: (i) consider a machine learning (ML) technique, which is a subfield of artificial intelligence, to clarify whether CRT or RT alone can lead to better disease-specific survival (DSS) outcomes in older patients with non-surgically treated stage III-IV HNSCC; (ii) evaluate the efficacy of CRT versus RT in terms of DSS using Kaplan-Meier (KM) survival curves and Cox proportional hazards model; and (iii) perform a propensity score-matching (PSM) analysis, which is an important statistical method for evaluating if a significant difference exists between older patients with non-surgically treated stage III-IV HNSCC that received either CRT or RT alone. The resulting analysis may aid in determining whether CRT offers a significant benefit over RT alone for older patients with advanced-stage HNSCC who are not candidates for surgery. To the best of our knowledge, no ML technique has previously been used to evaluate the effectiveness of CRT or RT in older patients with non-surgically treated advanced-stage HNSCC.

## Materials and Methods

### Collection of Data

**Fig. 1:**
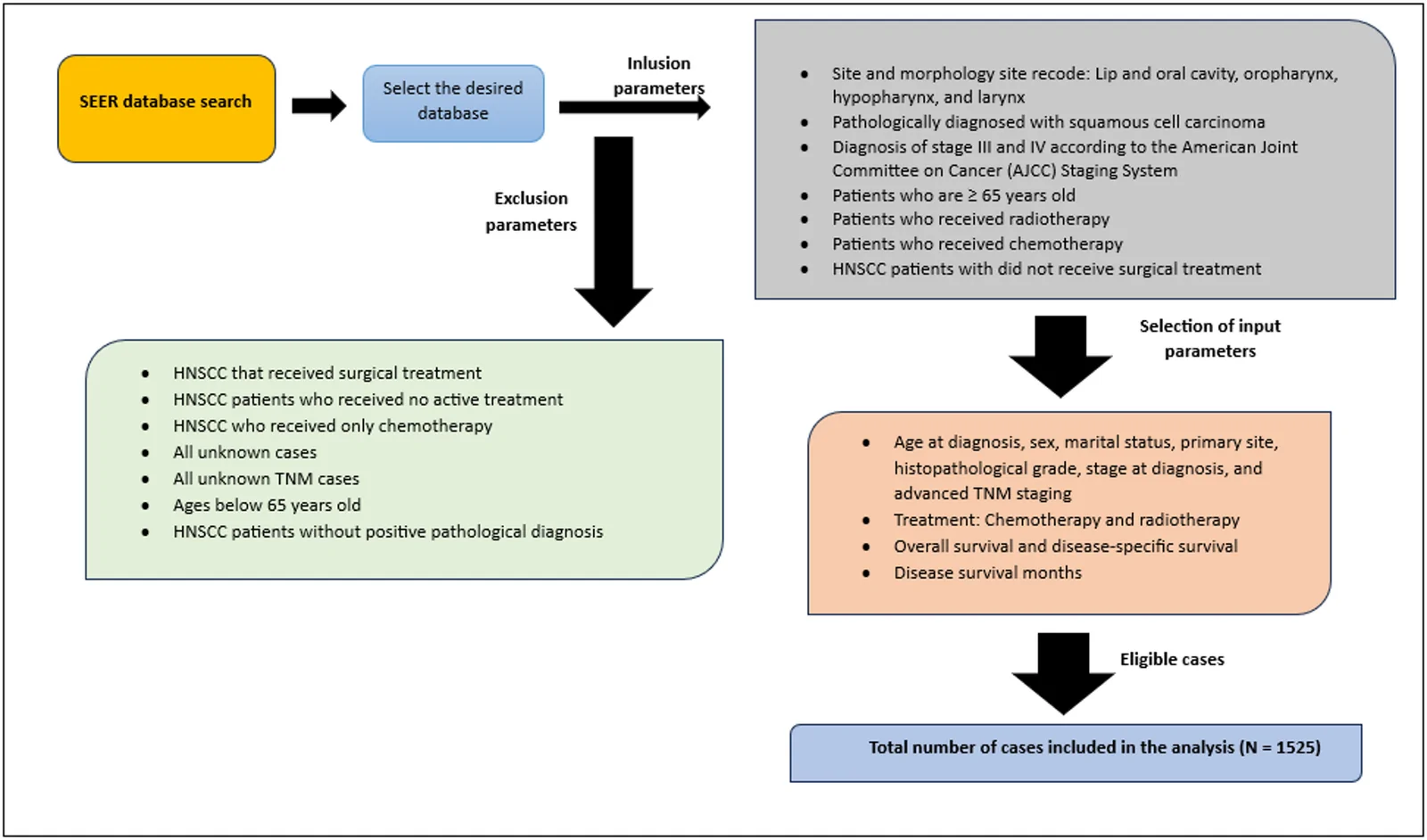
Data extraction process from SEER database

The data used in this study were extracted from the National Cancer Institute (NCI) through the Surveillance, Epidemiology, and End Results (SEER) Program of the National Institutes of Health (NIH). The extraction process is given in Fig. 1. The available HNSCC sites are presented in Table 1. In summary, these sites include lip and oral cavity, oropharynx, hypopharynx, and larynx. Owing to the objectives of the study, all the patients included in these sites: (a) were diagnosed with squamous cell carcinoma, (b) had not received surgery, (c) had received RT or CRT, and (d) were older than or equal to 65 years. The age threshold was set at 65 years, based on recent publications [1, 6, 16]. Additionally, a recent report by the World Health Organization (WHO) stated that the number of people aged 60 years and above outnumbered children younger than 5 years. Remarkably, the pace of ageing population in recent years is.

faster than in the past and will likely double from 12% to 22% based on recent projection [17]. All cases with missing data and cases where HNSCC was neither the first nor the only primary malignant tumor, were excluded. Ethical permission to use the SEER data was granted to the first author.

### Selection of Patients Attributes

Due to the limitations of the SEER database, the selected attributes include three main components – demographic, clinicopathologic, and treatment parameters. The demographic parameters include age at diagnosis, sex, and marital status, while the clinicopathologic parameters include the tumor nodal metastasis (TNM) status according to the American Joint Committee on Cancer (AJCC) Staging Scheme 7th edition, tumor site, tumor grade, and derived AJCC stage grouping (combined staging). Information on HPV status, frailty assessment, and comorbidity was lacking in the SEER database. The treatment parameters considered include RT alone or CRT (Table 1).

**Table 1:** Baseline demographic characteristics of HNSCC patients in the training data (*N* = 1525)

Variables	Total (*N* = 1525) (%)	Categorization for machine learning analysis	Data type after categorization
**Age at diagnosis**			
65–69 years old	593 (38.9%)	No categorization	Discrete
70–74 years old	400 (26.2%)		
75–79 years old	255 (16.7%)		
80–84 years old	150 (9.8%)		
$\:\ge\:$85 years old	127 (8.3%)		
**Sex**			
Female	304 (19.9%)	0 = Female	Categorical
Male	1221 (80.1%)	1 = Male.	
**Marital status**			
Married	825 (54.1%)	1 = Married	Categorical
Unmarried	700 (45.9%)	2 = Unmarried	
**Primary site**			
Lip and oral cavity	448 (29.4%)	0 = Lip and oral cavity (Primary Site 0)	Categorical
Oropharynx	297 (19.5%)	1 = Oropharynx (Primary Site 1)	
Hypopharynx	149 (9.8%)	2 = Hypopharynx (Primary Site 2)	
Larynx	631 (41.3%)	3 = Larynx (Primary Site 3)	
**Histopathological grade**			
Low grade (Well differentiated, Grade I and Moderately differentiated, Grade II)	966 (63.3%)	1 = Grade I	Categorical
High grade (Poorly differentiated, Grade III and Undifferentiated (Anaplastic), Grade IV	559 (36.7%)	2 = Grade II	
**Tumor stage**			
III	853 (55.9%)	III = 3	Categorical
IV	672 (44.1%)	IV = 4	
**T stage**			
T3	1525 (100.0%)	T3 = 3	
**N stage**			
N0	618 (40.5%)	N0 = 0	Categorical
N1	259 (17.0%)	N1 = 1	
N2	611 (40.1%)	N2 = 2	
N3	37 (2.4%)	N3 = 3	
**M stage**			
M0	1464 (96.0%)	M0 = Absent	Categorical
M1	61 (4.0%)	M1 = Present	
**Radiotherapy (RT)**			
RT alone	351 (23.0%)	0 = Only RT received	Categorical
**Chemoradiotherapy (CRT)**			
CRT alone	1174 (77.0%)	1 = Only CRT received	Categorical
**Disease-specific survival**			
Alive or dead of other causes	725 (47.5%)	0 = Alive or died due to other causes	Categorical
Dead of HNSCC	800 (52.5%)	1 = Dead of this cancer	

The distribution of these two treatment modalities, based on the considered sites and sex of advanced stage non-surgically treated HNSCC patients aged ≥ 65 years, is presented in Tables 2 and 3, respectively. The outcome of interest is the DSS of HNSCC patients (alive or died due to other cases or died of HNSCC).

**Table 2:** Each primary site and the corresponding treatment received

Primary site	RT	CRT
Lip and oral cavity	87 (24.8%)	361 (30.8%)
Oropharynx	58 (16.5%)	239 (20.4%)
Hypopharynx	38 (10.8%)	111 (9.5%)
Larynx	168 (47.9%)	463 (39.4%)
Total	351 (100.0%)	1174 (100.0%)

**Table 3:** Each primary site and the corresponding treatment for both sexes

Primary site	RT		CRT	
	**Male**	**Female**	**Male**	**Female**
Lip and oral cavity	58	29	299	62
Oropharynx	44	14	190	49
Hypopharynx	29	09	96	15
Larynx	142	26	363	100
Total	273 (77.8%)	78 (22.2%)	948 (80.8%)	226 (19.2%)

### Data Processing

Following the inclusion and exclusion criteria defined in **sub-**Sect. "Collection of data", a total of 1525 patients aged ≥ 65 years with stage III-IV HNSCC were included in this study (as they were not deemed to be candidates for surgery). The extracted features were categorized for machine learning and PSM analyses (Table 1). For the categorization, tumor stage III was included while stage IV included IVA, IVB, IVC, and IV NOS. For histopathological grading, well differentiated (grade I) and moderately differentiated (grade II) were categorized as low grade, while poorly differentiated (grade III) and undifferentiated (anaplastic, grade IV) were categorized as high grade. Marital status was recorded as married (including common law) or unmarried (single, separated, divorced, and widowed). In addition, according to the 7th AJCC staging system, stage IV was subcategorized into stages IVA, IVB, and IVC. In terms of TNM staging, advanced T stage included T3 and T4 (T4a, T4b, T4NOS), N stage included N0, N1, N2 (N2a, N2b, N2c, N2NOS), and N3, while M stage included M0 and M1.

### Patient Characteristics

The 1525 older patients extracted with stage III-IV HNSCC included 1221 males and 304 females in a male-to-female ratio of 4:1. The mean age at diagnosis was 73.1 years (SD $\:\pm\:\:$6.7: range 65–90) and the median age 72.0 years. In terms of older age categorization, 993 (65.1%) were younger old (ages 65–74 years), 405 (26.6%) were old (ages 75–84 years), and 127 (8.3%) were oldest old (≥ 85 years). The HNSCC sites in the series were lip and oral cavity (29.4%), oropharyngeal (19.5%), hypopharynx (9.8), and larynx (41.8%) (Table 1). Considering tumor stage, the AJCC 7th TNM staging scheme showed that all the patients had had stage T3. Most of the cases (40.5%) had N0 nodal classification. Specifically, in terms of the nodal parameter, 618 (40.5%) were N0, 259 (17.0%) were N1, 611 (40.1%) had N2, and 37 (2.4%) were N3, while 1464 (96.0%) had M0 and 61 (4.0%) M1. Regarding histopathological grading, 966 (63.3%) tumors were categorized as low grade (well-differentiated and moderately differentiated) and 559 (36.7%) were high grades (poorly differentiated and undifferentiated). The clinicopathologic characteristics are briefly summarized in Table 1.

In terms of marital status, 825 (54.1%) were married (including common law) while 700 (45.9%) were considered unmarried (single, divorced, widowed, and separated) at the time of diagnosis (Table 1). Considering the objective of this study in terms of oncological primary treatment, a total of 1174 (77.0%) patients received CRT while a total of 351 (23.0%) received RT alone. The details of the treatment received (CRT or RT alone) based on each primary site are presented in Table 2. The follow-up time ranged from 0 to 167 months (mean 48.8; median 29; SD $\:\pm\:\:$45.8), with a total of 725 (47.5%) patients being alive or died of other causes at the end of the follow-up.

### PSM

**Fig. 2:**
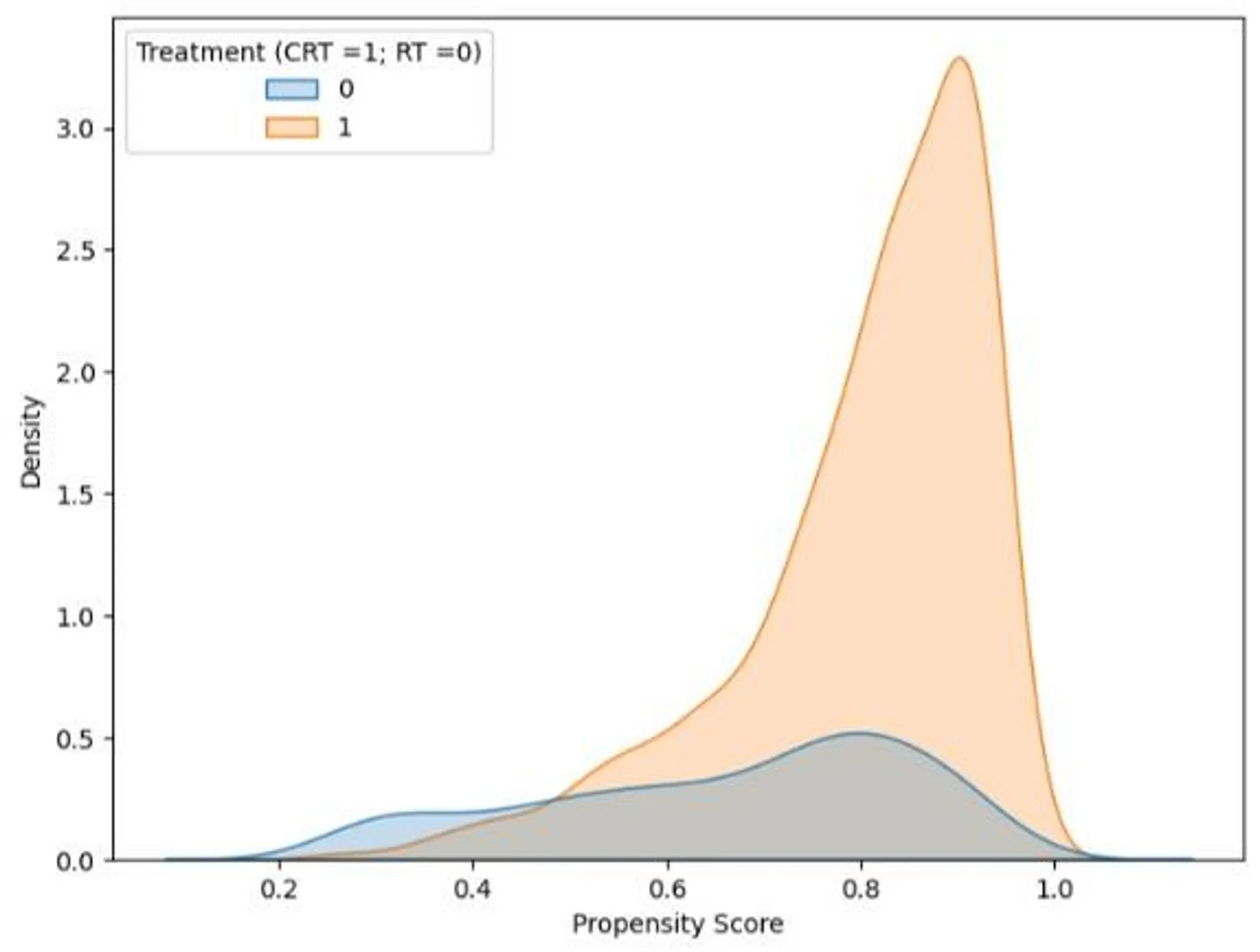
Distribution of estimated propensity scores according to treatment modality

The PSM was performed to reduce treatment-selection bias between patients receiving CRT or RT alone. Propensity scores were estimated using logistic regression incorporating the variables mentioned in sub-Sect. "Selection of patients attributes". Patients were matched 1:1 using nearest-neighbor matching without replacement based on the estimated propensity score. Covariate balance after matching was assessed using standardized mean differences. KM survival analyses and Cox proportional hazards regression were subsequently performed in the matched cohort to evaluate the association between treatment modality and DSS (Fig. 2).

### Kaplan Meier Survival Curves and Cox Proportional Hazards Model

**Fig. 3:**
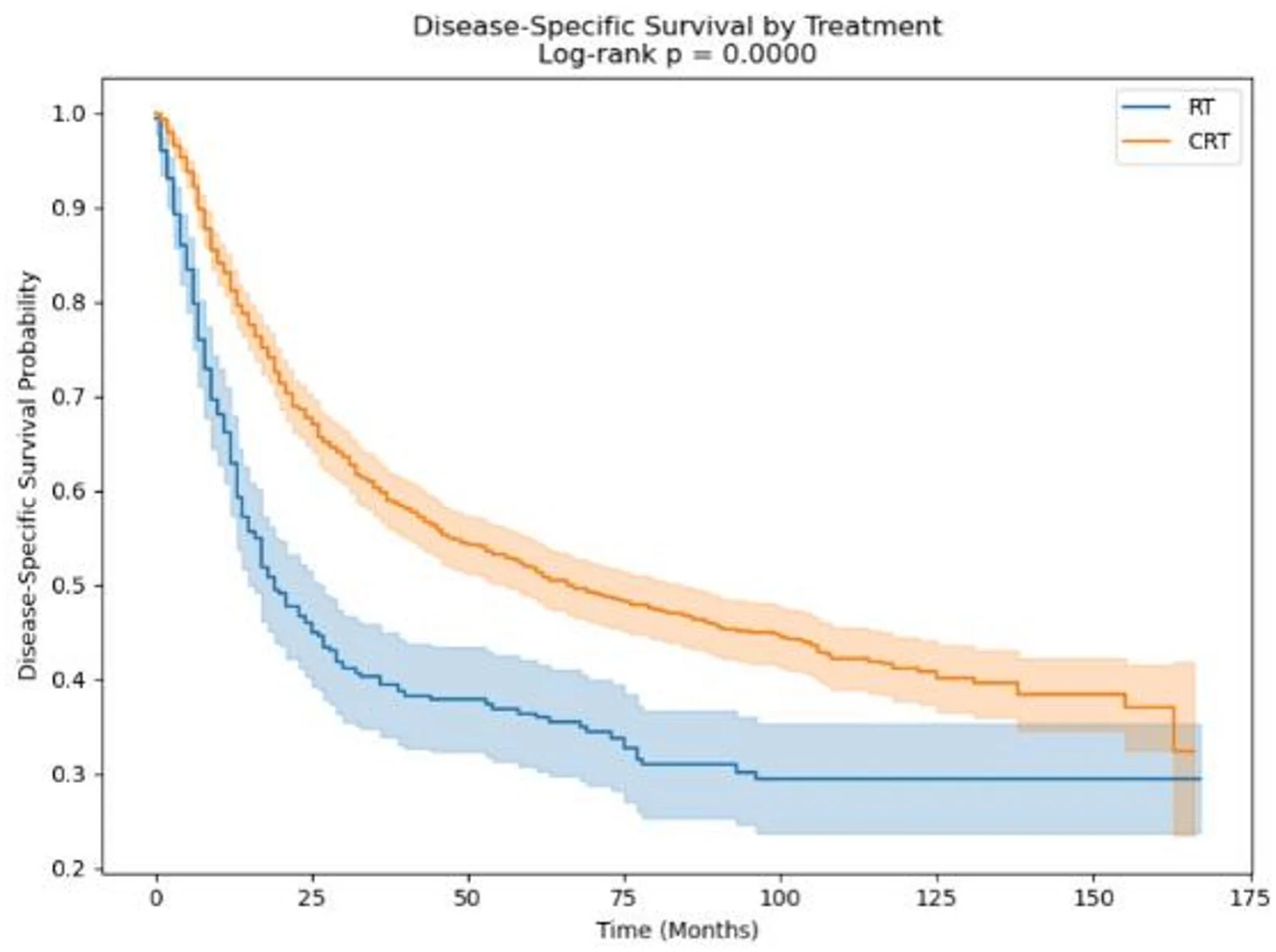
Kaplan-Meier curves for DSS stratified by treatment modality. Patients treated with concurrent CRT demonstrated significantly improved DSS compared with those treated with RT (log-rank *p* < 0.001). Shaded regions represent 95% confidence intervals

**Fig. 4:**
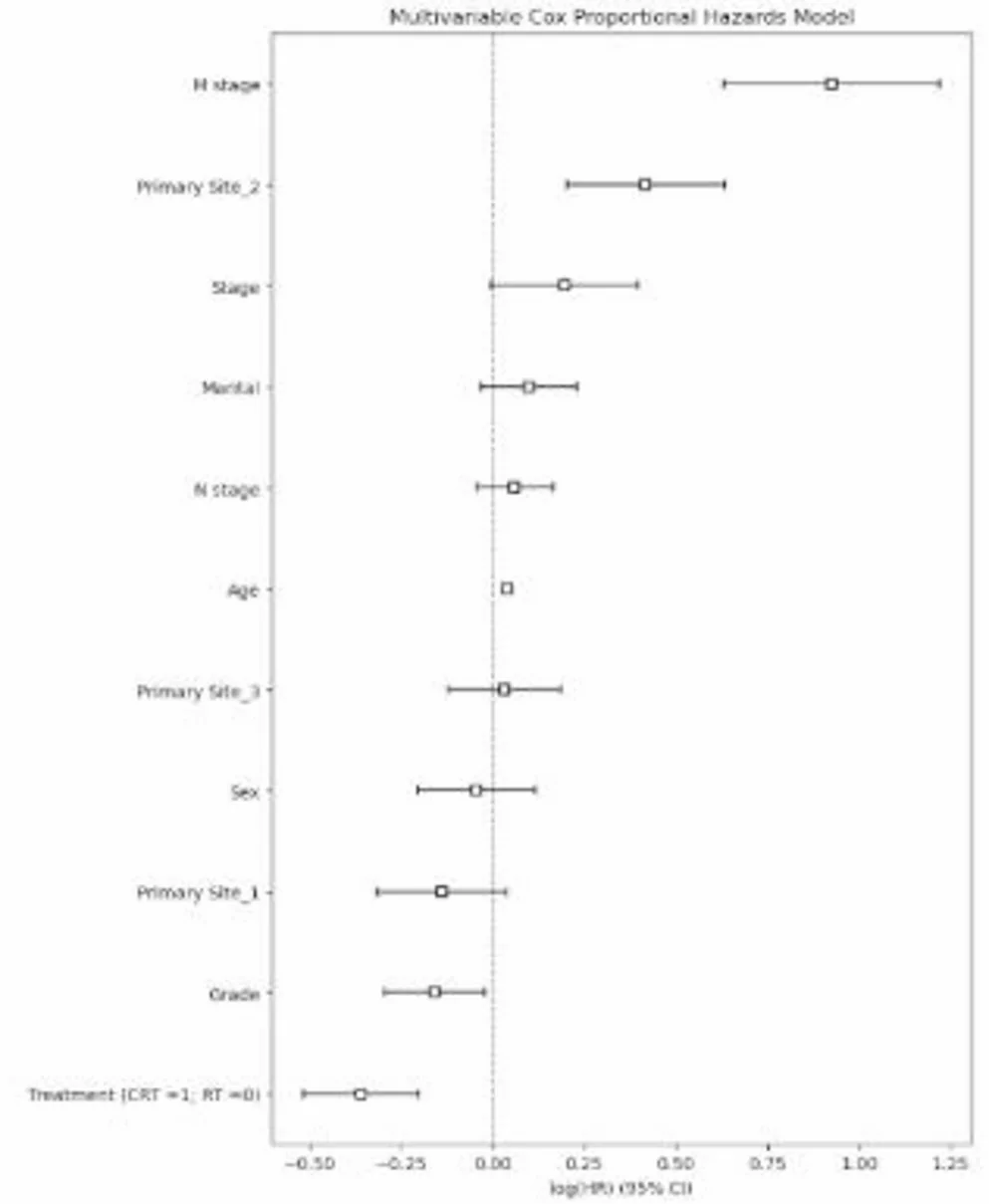
Forest plot of adjusted hazard ratios from the multivariable Cox proportional hazards model evaluating factors associated with DSS. Hazard ratios < 1 indicate a protective effect, whereas hazard ratios > 1 indicate an increased risk of disease-specific mortality. CRT was independently associated with improved DSS compared with RT alone (HR 0.70, 95% CI 0.60–0.82; *p* < 0.001). Legend: Primary Site labels in Table 1

In our study, we evaluated the DSS by using KM survival analysis and multivariable Cox proportional hazards regression. DSS was defined as the time from diagnosis to cancer-specific death, with patients who were alive at last follow-up were censored at the date of their last follow-up or who died from causes other than cancer treated as censored observations. Survival time was measured in months. The primary exposure of interest was treatment modality, categorized as RT (reference group) or concurrent CRT. Unadjusted survival outcomes were estimated using the KM method and compared between treatment groups using the log-rank test. KM survival curves were generated to visualize differences in DSS over time, with corresponding 95% confidence intervals (Figs. 3 and 4). The log-rank test was used to assess statistical significance between survival distributions.

**Fig. 5:**
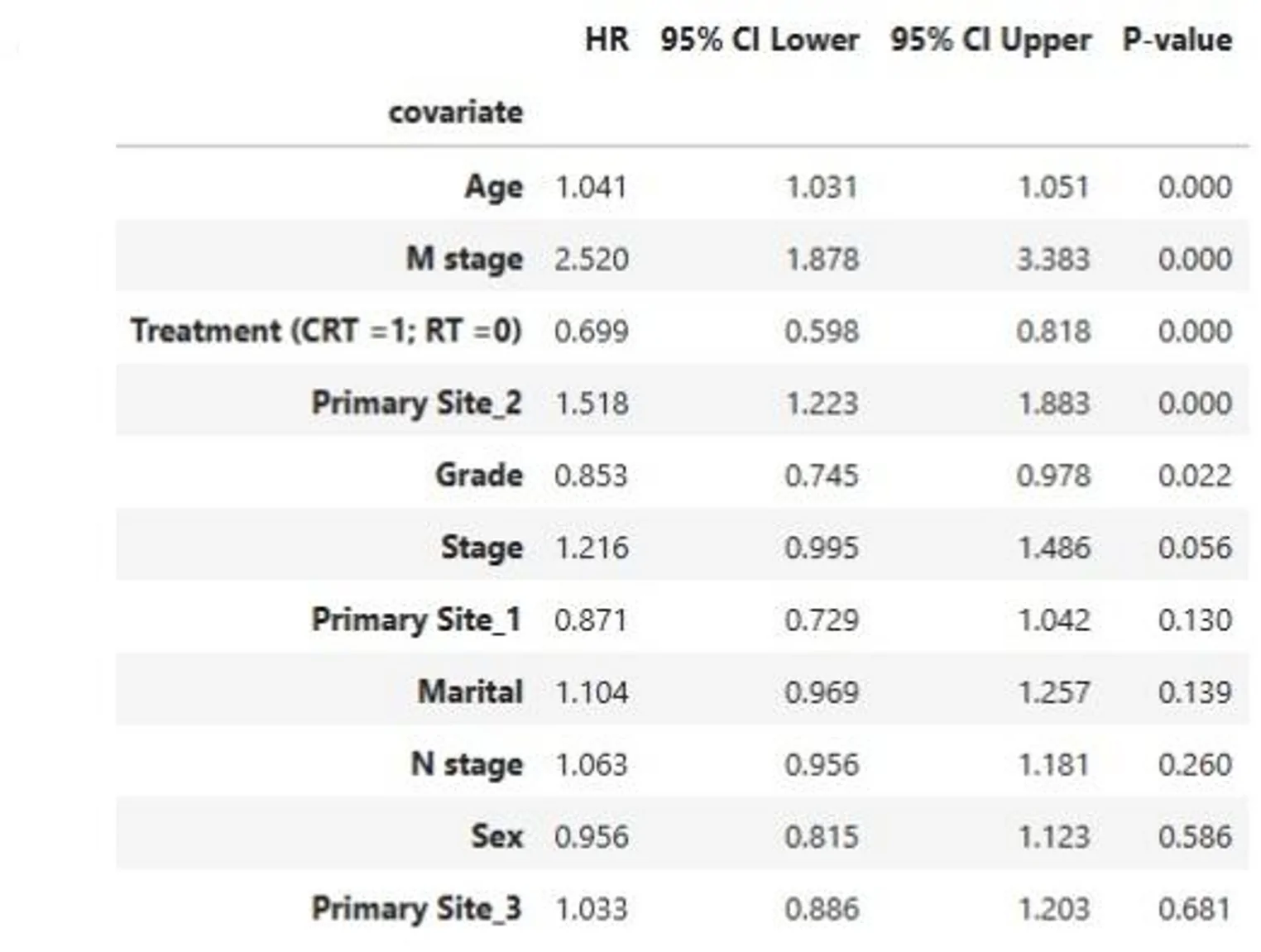
Multivariable Cox proportional hazards analysis of factors associated with DSS

To evaluate the independent effect of treatment while controlling for potential confounding factors, a multivariable Cox proportional hazards regression model was constructed. Covariates entered into the model are already described in Sub-Sect.  2.2. Age was modeled as a continuous variable, whereas primary site was treated as a categorical variable using dummy indicator variables. Variables exhibiting no variation across the study cohort were excluded before model fitting to ensure model convergence and stability. A penalized Cox regression approach was applied to address potential multicollinearity among covariates. Hazard ratios (HRs) and 95% confidence intervals (CIs) were reported, with HRs < 1 indicating a reduction in the hazard of disease-specific death and HRs > 1 indicating an increased hazard. Model assumptions were evaluated using standard diagnostic procedures for proportional hazards regression. Results from the Cox model were presented as adjusted hazard ratios and visualized using a forest plot. The combined use of KM survival curves and multivariable Cox regression enabled assessment of both unadjusted survival differences and the independent prognostic effect of treatment after adjustment for demographic and clinicopathological factors (Figs. 3 - 5) 

### Machine Learning Model Training

The model development process is given in Supplementary Figure I. The dataset for training the model consisted of (a) demographic parameters – age at diagnosis, sex, race, and marital status; (b) clinicopathological and tumor parameters – tumor site, tumor stage, tumor grade, and TNM staging; and (c) treatment parameters – RT and CRT. The outcome of interest was DSS.

The extracted data (**sub-**Sect. "Data processing") of 1525 HNSCC patients aged ≥ 65 years with non-surgically treated stage III-IV disease were imported into the Azure machine learning studio and also in Jupyter notebook. Following the loading of data, the necessary training parameters – selection of algorithms (extreme gradient boosting, logistic regression, and easy ensemble), learning rate, k-fold cross-validation (5-fold cross-validation), and performance metrics were defined. Various training hyperparameters were tuned to maximize the performance of the model. Due to the lack of an external independent geographic validation cohort in this study, we chose to implement a cross-validation method, which serves as a mini-external validation approach [18]. In this method, we repeatedly split the dataset into training and testing sets. Specifically, the data are divided into ‘k = 5’ folds, where the model is trained on k-1 folds and tested on the remaining fold. This process is repeated ‘k’ times. We adopted this approach to improve the predictive performance and generalizability of the model, while also minimizing the risk of overfitting by evaluating the model’s performance on multiple, distinct data subsets [18].

**Fig. 6:**
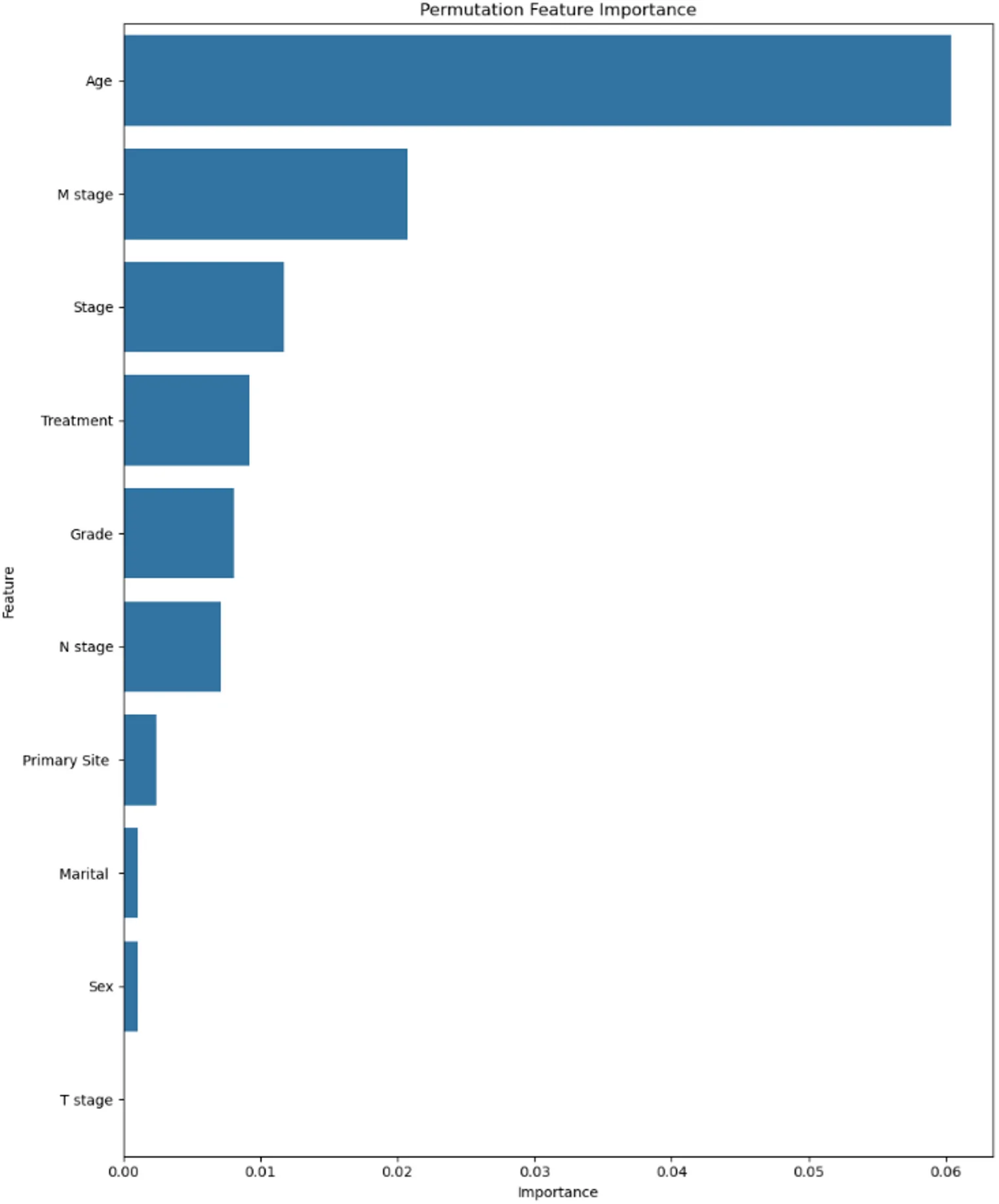
Aggregate feature importance of input variables for DSS prediction older patients with advanced-stage HNSCC not deemed suitable for surgery (Treatment option [CRT or RT alone])

The model was evaluated primarily based on performance accuracy. Other secondary metrics used were Sensitivity, Specificity, and area under receiving operating characteristics curve (AUC). We used feature importance analysis to evaluate the efficacy of treatment option (CRT or RT alone) in older patients with non-surgically treated stage III-IV HNSCC as an important input feature in (i) all the included HNSCC sites in this study; (ii) only oropharyngeal squamous cell carcinoma (OPSCC) patients (Supplementary material; Table 4); and (iii) only laryngeal squamous cell carcinoma (LSCC) patients (Supplementary material; Table 5). We used the Permutation Feature Importance (PFI) technique in the feature importance analysis. PFI is a feature ranking method that draws inspiration from the randomization techniques utilized in random forests (Fig. 6). As a model-agnostic approach, PFI is applicable to both classification and regression models, making it a versatile tool for assessing feature significance across various predictive modeling contexts. The PFI methodology involves a systematic process where features are randomly shuffled, one at a time (Fig. 6). The performance of the model is evaluated using predefined performance metrics, such as accuracy, both prior to and following the permutation of features. The resulting scores from this process reflect the change in the model’s performance attributable to the shuffling of each feature. Features deemed important are typically more sensitive to this shuffling process, leading to higher importance scores.

Essentially, a PFI score quantifies the contribution of a specific feature to the predictive performance of the model, providing insights into its relevance. In contrast to other feature selection techniques, such as Filter-Based Feature Selection, which compute scores based on the characteristics of the data independent of the model, PFI evaluates the model’s performance before and after the reshuffling of features. This post-hoc analysis allows for a more nuanced understanding of how each feature influences the model’s predictions, thereby enhancing the interpretability of the model and guiding feature selection in subsequent modeling efforts. By focusing on the actual impact of features on model performance, PFI serves as a robust method for identifying critical predictors in complex datasets. We have carefully selected PFI in this study mainly because PFI doesn’t measure the association between a feature and a target value. Instead, it captures how much influence each feature has on predictions from the model.

**Table 4:** Baseline demographic characteristics of oropharyngeal squamous cell carcinoma patients in the training data (*N* = 297)

Variables	Total (*N* = 297) (%)	Categorization for machine learning analysis	Data type after categorization
Age at diagnosis			
65–69 years old	115 (38.7%)	No categorization	Discrete
70–74 years old	81 (27.3%)		
75–79 years old	56 (18.9%)		
80–84 years old	26 (8.8%)		
$\:\ge\:$85 years old	19 (6.4%)		
**Sex**			
Female	63 (21.2%)	0 = Female	Categorical
Male	234 (78.8%)	1 = Male.	
**Marital status**			
Married	163 (53.6%)	1 = Married	Categorical
Unmarried	134 (46.4%)	2 = Unmarried	
**Tumor grade**			
Low grade (Well differentiated, Grade I and Moderately differentiated, Grade II)	151 (50.8%)	1 = Grade I	Categorical
High grade (Poorly differentiated, Grade III and Undifferentiated (Anaplastic), Grade IV	146 (49.2%)	2 = Grade II	
**Tumor stage**			
III	118 (39.7%)	III = 3	Categorical
IV	179 (60.3%)	IV = 4	
**T stage**			
T3	297 (100.0%)	T3 = 3	Categorical
**N stage**			
N0	67 (22.6%)	N0 = 0	Categorical
N1	54 (18.2%)	N1 = 1	
N2	163 (54.9%)	N2 = 2	
N3	13 (4.4%)	N3 = 3	
**M stage**			
M0	289 (97.3%)	M0 = Absent	Numeric
M1	08 (2.7%)	M1 = Present	
**Radiotherapy (RT)**			
RT alone	58 (19.5%)	0 = Only RT received	Numeric
**Chemoradiotherapy (CRT)**			
CRT alone	239 (80.5%)	1 = Only CRT received	
**Disease-specific survival**			
Alive or dead of other causes	150 (50.5%)	0 = Alive at the end of follow-up	Numeric
Dead of HNSCC	147 (49.5%)	1 = Dead of this cancer or other causes	

**Table 5:** Baseline demographic characteristics of laryngeal squamous cell carcinoma patients in the training data (*N* = 631)

Variables	Total (*N* = 631) (%)	Categorization for machine learning analysis	Data type after categorization
**Age at diagnosis**			
65–69 years old	250 (39.6%)	No categorization	Discrete
70–74 years old	173 (27.4%)		
75–79 years old	108 (17.1%)		
80–84 years old	51 (8.1%)		
$\:\ge\:$85 years old	49 (7.8%)		
**Sex**			
Female	126 (20.0%)	0 = Female	Numeric
Male	505 (80.0%)	1 = Male.	
**Marital status**			
Married	319 (50.6%)	1 = Married	Numeric
Unmarried	312 (49.4%)	2 = Unmarried	
**Tumor grade**			
Low grade (Well differentiated, Grade I and Moderately differentiated, Grade II)	466 (73.9%)	0 = Grade I	Numeric
High grade (Poorly differentiated, Grade III and Undifferentiated (Anaplastic), Grade IV	165 (26.1%)	1 = Grade II	
**Tumor stage**			
III	463 (73.4%)	III = 3	Numeric
IV	168 (26.6%)	IV = 4	
**T stage**			
T3	631 (100.0%)	T3 = 3	Numeric
**N stage**			
N0	384 (60.9%)	N0 = 0	Numeric
N1	92 (14.6%)	N1 = 1	
N2	147 (23.3%)	N2 = 2	
N3	08 (1.3%)	N3 = 3	
**M stage**			
M0	606 (96.0%)	M0 = Absent	Numeric
M1	25 (4.0%)	M1 = Present	
**Radiotherapy (RT)**			
RT alone	168 (26.6%)	0 = Only RT received	Numeric
**Chemoradiotherapy (CRT)**			
CRT alone	463 (73.4%)	1 = Only CRT received	
**Disease-specific survival**			
Alive or dead of any other causes	309 (49.0%)	0 = Alive at the end of follow-up	Numeric
Dead of this cancer	322 (51.0%)	1 = Dead of this cancer or other causes	

### Shapley Additive Explanations

**Fig. 7:**
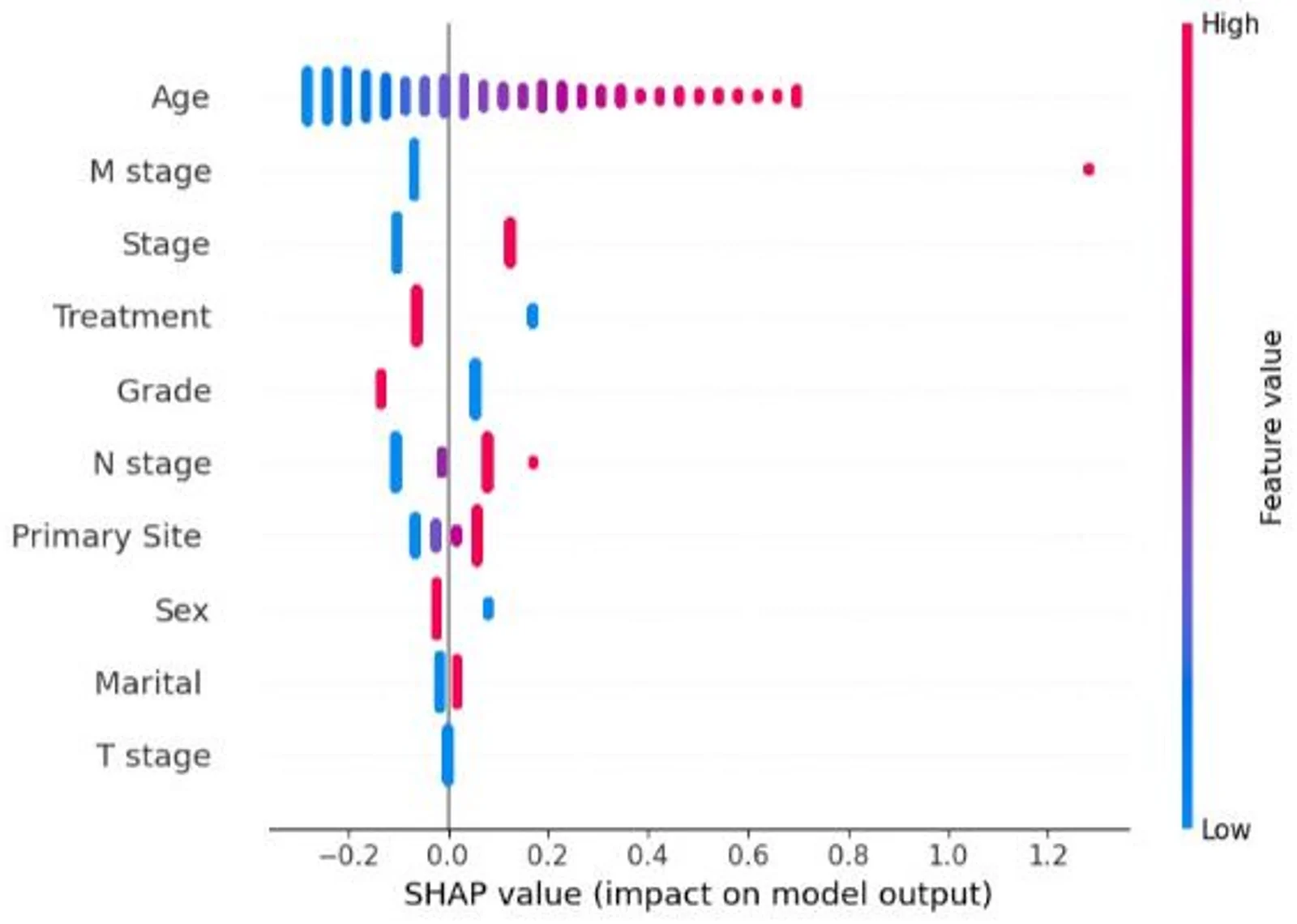
SHAP analyses for explainability and interpretability

**Fig. 8:**
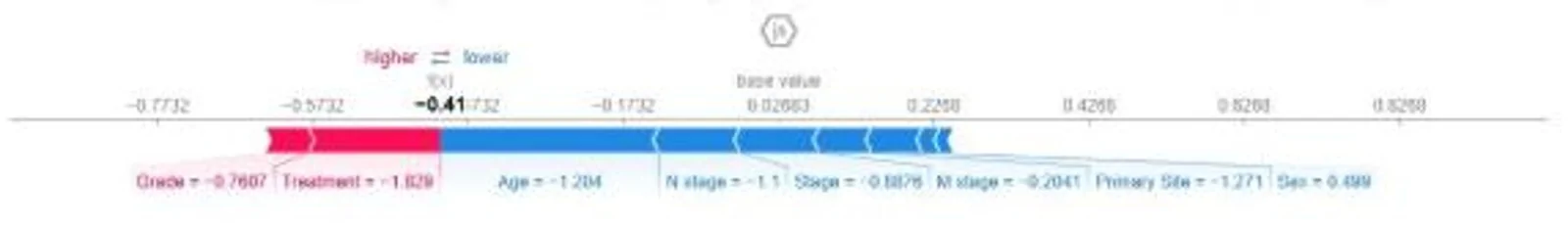
Waterfall plot illustrating the SHAP values for an individual patient. Red bars indicate variables increasing the predicted probability of disease-specific death, whereas blue bars indicate variables decreasing the predicted probability. The magnitude of each SHAP value reflects the relative contribution of the corresponding feature to the final prediction

We used SHapley Additive exPlanations (SHAP) to provide interpretations to the predictions made by the best-performing ML model. SHAP works by quantifying the contribution of each predictor variable to an individual prediction by assigning a Shapley value that is based on concepts of cooperative game theory. The SHAP TreeExplainer was applied to the final XGBoost model using the tree_path_dependent feature perturbation approach. The global model interpretability was assessed using a SHAP beeswarm plot, which displays the distribution and magnitude of feature effects across all patients and identifies the most influential predictors (Fig. 7). Local model interpretability was evaluated using SHAP waterfall plots, which illustrate how individual features contributed to a specific prediction by increasing or decreasing the predicted probability of DSS (Fig. 8). Features with larger absolute SHAP values were considered to have a greater impact on model predictions.

## Results

### PSM Analysis

The balance between the entire cohort after matching showed substantial differences in treatment allocation between patients receiving RT and concurrent CRT (Fig. 2).

The estimated propensity score distributions showed substantial overlap between treatment groups, indicating the presence of a common support region suitable for matching. However, patients treated with CRT generally exhibited higher propensity scores and a more concentrated distribution compared with those receiving RT, suggesting that baseline demographic and clinicopathological characteristics influenced treatment selection (Fig. 2). Despite these differences, the overlapping propensity score distributions support the feasibility of propensity score matching to reduce treatment-selection bias and improve comparability between treatment cohorts. These findings justify the use of propensity score matched analyses to more accurately estimate the independent effect of CRT versus RT on DSS while minimizing the impact of measured confounding variables.

### Kaplan Meier Survival Curve

The KM analysis demonstrated a significant survival advantage for patients treated with CRT compared with RT alone (Fig. 3).

The survival curves separated early and remained consistently distinct throughout the follow-up period, indicating a durable treatment effect. Patients receiving CRT exhibited substantially higher DSS probabilities across the entire follow-up interval, whereas the RT group experienced a more rapid decline in survival. The difference between treatment groups was highly significant according to the log-rank test (*p* < 0.001).

### Cox Proportional Hazards Model

Our multivariate Cox proportional hazards regression analysis demonstrated that treatment modality was independently associated with DSS.

As shown in Fig. 4, treatment with concurrent CRT was associated with a significantly lower hazard of disease-specific death compared with RT alone, demonstrating the strongest protective effect among all variables included in the model. The regression coefficient for treatment was negative, indicating a substantial reduction in mortality risk among patients receiving CRT. Conversely, the presence of distant metastasis emerged as the most powerful adverse prognostic factor, exhibiting the largest positive effect on the hazard of disease-specific death. Increasing overall stage was also independently associated with worse survival outcomes. Primary tumor site demonstrated heterogeneous prognostic effects, with Primary Site 2 (hypopharynx) associated with an increased hazards (disease-specific death) relative to the reference site (lip and oral cavity), whereas Primary Site 1 (oropharynx) appeared to confer a DSS advantage. Primary Site 3 (LSCC) shares similar DSS with the reference site (Fig. 4).

N stage showed a modest adverse association with DSS, while age contributed a small but measurable increase in mortality risk. Sex, marital status, Primary Site 3 (LSCC), and tumor grade demonstrated comparatively weaker effects, with confidence intervals overlapping the null value, suggesting limited independent prognostic significance after adjustment for other clinicopathological factors.

As shown in our multivariable Cox proportional hazards regression analysis (Fig. 5), M stage (HR 2.520, 95% CI 1.878–3.383, *p* < 0.001), Primary Site 2 (hypopharyngeal cancer) (HR 1.518, 95% CI 1.223–1.883, *p* < 0.001), and increasing age (HR 1.041, 95% CI 1.031–1.051, *p* < 0.001) were independently associated with poorer DSS. Conversely, CRT was associated with a significantly reduced risk of disease-specific mortality compared to RT alone (HR 0.699, 95% CI 0.598–0.818, *p* < 0.001), corresponding to an approximately 30% reduction in the hazard of cancer-specific death. Tumor grade was also significantly associated with survival (HR 0.853, 95% CI 0.745–0.978, *p* = 0.022) (Fig. 5).

Adjusted hazard ratios (HRs), 95% confidence intervals (CIs), and corresponding *p*-values derived from the multivariable Cox proportional hazards regression model evaluating predictors of DSS. Legend: Primary Site labels in Table 1.

Other related figures showing the multivariable Cox proportional hazards analysis of factors associated with DSS are presented in Supplementary Figure II. Specifically, the multivariable Cox proportional hazards analysis of factors associated with DSS for OPSCC and LSCC are presented in Supplementary Figures III – X.

### ML Performance Accuracy and PFI

The performance accuracy of the extreme gradient boosting (XGB) algorithm was 55.5% for estimating DSS in older patients with non-surgically treated stage III-IV HNSCC. The precision, recall, F1 score, and Area Under receiver operating characteristics Curve (AUC) were 0.58, 0.56, 0.57, and 0.57. In terms of importance of input features for DSS in this subgroup, the top four predicted features were age, M stage, overall TNM stage, and treatment option (CRT or RT alone) (Fig. 6).

The PFI analysis for OPSCC and LSCC are presented in Supplementary Figures XI – XII. The contribution of each of these input features is explained using SHAP (sub-Sect. "Model interpretability and explainability using SHAP").

### Model Interpretability and Explainability using SHAP

The ML-based explainability SHAP analyses corroborated the survival modeling results. SHAP analysis demonstrated that age was the most influential predictor of model output, followed by M stage, overall TNM stage, treatment modality, tumor grade, and N stage (Fig 7).

Higher age, advanced stage, and M stage were associated with increased predicted mortality risk, whereas treatment with CRT contributed substantially toward improved survival predictions (Fig. 7). Similarly, being married contributed to better DSS. This represents the overall global explanation. A similar explanation can be used for an instance of a single patient (Fig. 8). A local SHAP (a single instance) analysis was performed to interpret an individual prediction generated by the final machine learning model (Fig. 8). The model predicted a lower probability of disease-specific death for this patient (SHAP output, *f(x)* = -0.42 relative to a baseline risk of 0.03). Among the evaluated predictors, treatment status and tumor grade were the strongest contributors toward reducing the predicted risk, whereas sex was the principal factor increasing the predicted risk. Additional variables, including age, N stage, overall stage, M stage, and primary tumor site, further contributed to lowering the predicted probability of disease-specific mortality.

Collectively, these findings demonstrate how individual clinical and tumor-related characteristics interact to influence patient-specific risk estimations and highlight the ability of the model to provide transparent and clinically interpretable predictions.

## Discussion

This study, to the best of our knowledge, represents the first study using an ML technique to compare the effectivness of CRT versus RT alone for older patients with stage III-IV HNSCC not deemed to be candidates for surgery. We demonstrate that, in the SEER database cohort, CRT was associated with better disease-specific survival (DSS) than RT alone. The ML model we developed examined the complex relationships between demographic, tumor, clinicopathologic, and treatment parameters by analyzing how HNSCC patients aged ≥ 65 years with stage III-IV disease with histologically confirmed squamous cell carcinoma benefit from either CRT or RT alone. Although our ML model did not show significant potential to predict DSS for new patients under management consideration, the PFI results fulfilled our ultimate goal to aid in personalized treatment planning for older patients with HNSCC by providing how the input features contributed to DSS.

Individualized therapeutic decision-making based on survival prognostication remains one of the most difficult challenge in the management of older patients with HNSCC [6]. This is due to the complex and heterogeneous nature of the disease itself and also to patient- and treatment-related factors. With the aging population and a rising incidence of HNSCC, there is an emerging need to develop strategies for the management of older patients [6]. This is particularly important owing to the comorbidities associated with many of these patients. Therefore, this subgroup of HNC patients usually needs complex evaluation and pretreatment management. Due to this, older HNC patients may often be underrepresented in clinical trials. Consequently, there is a dearth of scientific evidence to determine whether older HNC patients constitute the highest-risk groups regarding treatment-related morbidity and mortality, and if they can tolerate and benefit from aggressive treatment.

This study leverages the ML paradigm for enhanced prediction of DSS for older patients with HNSCC. The model was developed using the voting and stacked ensemble methods, and these models showed promising performance. The feature importance analysis showed that treatment option (CRT) represents one of the four most important parameters for DSS in older patients with stage III-IV HNSCC. This finding is in agreement with several randomized controlled trials studies concluding that CRT may result in better DSS than RT alone [19–21]. Similarly, in recent years, other studies have explored various statistical methods such as propensity score matching and Cox regression models to evaluate the efficacy of CRT over RT alone among older patients with advanced-stage HNC [1].

Choosing between treatment options in older patients is often challenging, and careful selection of the treatment option is recommended. This is a frequent scenario in clinical practice, as 35–40% of patients with HNSCC are older. Of note, surgical procedures for older patients with HNSCC may increase the risk of morbidity and mortality in some patients within this subgroup [22, 23]. In addition, recovery from surgery and functional deficits due to surgery may make surgery unsuitable for older patients with significant comorbidities. Therefore, older patients were far more commonly treated with nonsurgical methods, such as CRT or RT alone, in the SEER database used in this study [24]. As a result, exploring other optimal treatment strategies for this subgroup is warranted. Intriguingly, experience has shown that older patients with HNSCC may withstand intensive, curative treatment without experiencing a more pronounced decline in their quality of life [6]. However, in terms of optimal treatment strategies, conflicting reports have been published regarding the efficacy of CRT and RT alone [1, 6]. Some studies emphasized the efficacy of CRT over RT alone [19, 25] while other studies have reported that RT alone resulted in better OS than CRT [21]. As such, the interdisciplinary tumor-board has a significant role to play in deciding the best therapy for each patients [26].

For example, for OPSCC, our feature importance analysis showed that CRT represents one of the five most important features for DSS. This finding corroborates the standard care recommendation for advanced stage OPSCC [26–28]. Despite this, there is a growing predilection for surgical approaches followed by adjuvant treatment [26]. Similarly, for LSCC, the debate regarding the ideal treatment for LSCC is old and still ongoing [29]. When the tumor is too expensive for effective organ preservation or when preservation would leave a nonfunctional larynx, the gold standard treatment for advanced-stage LSCC remains total laryngectomy. During the past decades, more conservative treatments and organ-preserving surgery have gained popularity [29]. This is in tandem with the results from the feature importance analysis in this study, which identified treatment option (CRT or RT alone) as one of the three most important features for DSS in older patients with stage III-IV HNSCC not deemed to be candidates for surgery. Of note, suboptimal treatment arising from low irradiation and low chemotherapy response may result in lower tumor response [29], consequently resulting in salvage laryngectomy.

Although this study has confirmed the survival benefit of CRT in treating HNSCC patients aged ≥ 65 years with III-IV-HNC not deemed to be candidates for surgery, the choice of chemotherapy-based treatment combined with RT should be carefully selected for the associated side effects. In a recent study by Shenoy et al., the authors concluded that either of these treatment approaches may lead to good outcomes and should be preferred to a palliative approach, especially for oral cavity cancer [30]. While most guidelines may tilt towards platinum-based chemotherapy, the use of the monoclonal antibody (with an epidermal growth factor receptor [EGFR] inhabitation effect) cetuximab plus RT offers less toxicity compared to cisplatin-based chemotherapy even though the latter was reported to show better OS than the former, especially for human papillomavirus-positive (HPV-positive) oropharyngeal cancer [31, 32].

Typically, for RT to yield the desired result, precise staging and effective tumor delineation remains an essential prerequisite. This is important since small errors, either in tumor definition or nodal extent can lead to either geographic miss of malignant tissue or unnecessary irradiation of healthy structures. While computed tomography (CT)-based planning remains the clinical standard, 18 F-fluorodeoxyglucose positron emission tomography/CT (18 F-FDG PET/CT) has been shown in recent work to offer measurable benefits for both staging and treatment planning [33]. In a recent study of patients with HNSCC undergoing radical volumetric modulated arc RT with concurrent cisplatin CT, it was found that PET/CT meaningfully altered TNM staging, increasing the assigned T stage in 11.8% of patients and the N stage in 33.3% of patients, and additionally identified distant metastatic disease in 9.8% of patients that had not been apparent on conventional imaging [33]. These staging changes are clinically consequential. This is particularly important since upstaging based on nodal or distant disease can shift a patient’s overall treatment intent, or prompt the inclusion of additional disease sites within the radiation field [33, 34].

Beyond staging, PET/CT-based imaging directly reshapes the physical radiotherapy plan itself. In a recent study, a comparative analysis involving planning target volumes (PTVs) for both the primary tumor and nodal regions were defined independently using CT alone and PET/CT by a single radiation oncologist, it was found that the resulting dose-volume histograms showed that PET/CT altered coverage of the primary tumor and identified high-risk neck regions that CT-based contouring alone had missed [33].

A further, more forward-looking benefit is the ability of FDG-PET to guide biologically informed dose distribution, an approach not possible with anatomical CT imaging alone. Because glycolytically active, hypermetabolic tumor regions tend to correspond to more hypoxic, radioresistant cell populations, identifying these regions on PET allows radiation oncologists to apply focal dose escalation, or “dose painting,” to the areas most likely to require a higher dose for local control. In patients with HNSCC, this translated directly into clinical practice through the administration of a booster focal dose to large lymph nodes exhibiting high metabolic activity on PET, a modification that would not have been identifiable using CT contours alone [33]. This capacity to tailor dose distribution to the underlying biological heterogeneity of the tumor, rather than to its anatomical boundaries alone, represents one of the more distinctive advantages of PET-CT-based planning over conventional approaches. Taken together, 18 F-FDG PETCT-based RT planning offers concrete benefits in HNSCC across at least three dimensions: more accurate staging, more precise and clinically appropriate target volume delineation, and the ability to biologically guide dose distribution through functional imaging [33, 34].

There are certain ethical, regulatory, and data related issues preventing the recommendation of ML-based models for further clinical evaluations [35–37]. Despite this, the last few years have witnessed various technological developments that can revolutionize the medical field. The Internet of Things (IoT) represents one of the most significant of these evolving technologies with potentials in clinical practice. A recent systematic review characterized the potential of IoT-enabled devices for telesurgery, surgical telemonitoring and telementoring, image-guided surgery, and postoperative patient telemonitoring [38]. This is particularly important in head and neck oncology since surgical management frequently involves microvascular free-flap reconstruction, a procedure in which early detection of vascular compromise is critical to flap survival and where conventional monitoring still relies heavily on intermittent, subjective clinical examination [38, 39]. In recent years, IoT-based monitoring has been posited as a viable approach to addressing this problem.

For example, a recent study used an implanted Doppler probe connected via Wi-Fi, thereby allowing surgical team to track flap perfusion remotely and continuously from any location, rather than relying solely on scheduled bedside checks [39]. This approach offered a higher flap survival and a shorter time to revision surgery in the IoT-monitored group compared with conventional monitoring [39]. Rather than replacing clinical judgement, this IoT-based approach serves as an ancillary tool for continuous remote data stream that has the capability to extend the window of surveillance beyond the moments a clinician is physically present. Thus, potentially allowing earlier detection of complications such as flap ischemia, airway compromise, or postoperative bleeding [38, 39]. As digital transformation continues to revolutionize many disciplines, the use of IoT-enabled sensors and connected monitoring platforms continues to mature, their integration into postoperative HNC is widely incorporated but less investigated concept. Advantages have become palpable over the past decade, yet further research is warranted [38].

Beyond the application of IoT in HNSCC oncology, three-dimensional (3D) printing has moved from a novelty into a genuine bridge between medical imaging, education, and clinical practice, converting patient-specific CT or MRI data into physical anatomical models and surgical devices via computer-aided design and additive manufacturing. This is particularly important in HNC oncology since tumor resection frequently requires complex, patient-specific bony reconstruction, most often of the mandible or maxilla, where small deviations in plate contouring can compromise both function and cosmesis. In a recent comparative study involving head and neck reconstruction, patients whose surgery used 3D-printed, patient-specific surgical plates achieved significantly more accurate bony reconstruction than those treated with conventionally bent plates, with a mean reduction in reconstruction deviation of 0.7 mm [40]. Therefore, 3D printing in HNC can draw directly on preoperative imaging data, supports surgical training and patient communication through tangible anatomical models, and translates into measurably more precise reconstructive practice at the operating Table [41].

The main strength of the present work is the exploration of an ML technique to analyze HNSCC patients aged ≥ 65 years treated exclusively with concurrent CRT or RT alone for tumors of a single histology. Despite this strength, our study has some limitations. It should be emphasized that the limitations observed in this study primarily reflect the inherent constraints of the SEER database, rather than deficiencies in the study design or cohort. First, the retrospective nature of the data extracted from SEER may introduce selection bias and limit the comprehensive collection of variables. This limitation is peculiar to the SEER database and retrospective studies in general. Second, there is considerable variation in the distribution of HNSCC sites in this study, with majority of the patients diagnosed with oropharyngeal or laryngeal cancer. This uneven distribution may increase the likelihood of analytical bias. Another limitation inherent to the SEER database is the lack of information about other important variables including interval between treatment completion and death, comorbidities, frailty screening, type of chemotherapy, completeness of chemotherapy and completeness, radiotherapy type (IMRT/VMAT or 3DCRT), total dose, and detailed dosimetric information. For instance, in the SEER database, chemotherapy was presented as a binary variable (administered versus not administered) without considering the nuances of the actual regimen. Considering the specific chemotherapy regimens, including dose and combination of drugs, can significantly impact a patient’s survival and other outcomes. Without the detailed information on the regimen, it may be challenging to accurately assess patient adherence to treatment guidelines.

Similarly, the lack of information on the radiation techniques or their purpose (palliative or curative) has been underreported in the SEER database. While there have been efforts to add more data on radiotherapy details, the information on radiotherapy for the SEER registry used for this study was not comprehensive. The lack of such complete data can introduce bias when analyzing associations between treatment and outcomes, as it may be impossible to link specific treatment types or their intent to the patients who received them. Furthermore, comorbidities have been found to significantly decrease survival in patients with advanced-stage cancer [42, 43]. This is because they can significantly limit treatment options, increase the risk of treatment-related complications, and worsen the patient’s overall health and performance status. The more severe or numerous a patient’s comorbidities, the lower their chance of survival. Also, it was neither possible to evaluate how many patients developed treatment-related complications nor to ascertain if the presence of comorbidities reduced the treatment efficacy in these patients.

Beyond comorbidity, frailty was another important parameter missing in the SEER database. This is particularly important since frailty has been considered as an important index that predicts DSS and toxicity in older patients [44]. Without detailed information on frailty, it may be challenging to assess patients who may be at risk for poor outcomes and, consequently, to remove them from the analysis to avoid bias in terms of the efficacy of treatment methodologies presented in this study.

The absence of information regarding the HPV status of the patients is another important limitation of this study, given that the HPV status is a key prognostic factor in OPSCC. The absence of this information substantially limits the interpretation of survival benefit for OPSCC patients presented in this study, since HPV-positive HNSCC is more sensitive to treatment and thus has more favorable survival than its HPV-negative counterpart [45]. Having information about the HPV status will be instrumental to the interpretation of the results presented in this study since HPV positivity was associated with significantly improved survival outcomes, suggesting that HPV-associated tumors maintain their favorable prognosis even in older OPSCC patients [46]. Most importantly, the presence of this information can further strengthen the argument on the efficacy of the examined treatment approaches in this study, potentially supporting strategies like treatment de-escalation for managing these tumors even in this age group. Additionally, variables such as quality of life of patients, which were missing in the SEER database, should be considered in future analysis due to the advanced age of the patients. At such age, it is important to ascertain whether death was caused by HNC or by other related comorbidities.

In conclusion, our model did not show promising performance accuracy in predicting the OS for older HNSCC patients with stage III-IV disease and not deemed to be candidates for surgery. However, our study provides an insight into a potential survival benefit for CRT in older patients with stage III-IV HNSCC who are not candidates for surgery. This further emphasizes that treatment decisions at an advanced stage may be made according to other patient-related factors rather than chronological (calendar) age alone. Despite the results obtained in this study, its interpretation should be considered exploratory only, due to the potential for analytical bias due to the imbalanced data used in this study. Therefore, multicenter, randomized controlled trials and age-specific prospective clinical studies are warranted to confirm the efficacy of CRT over RT alone for advanced-stage HNSCC, with separate analyses for different tumor sites. In further studies, the model developed may contain information about the type of chemotherapy and information about the completeness of chemotherapy to make it more robust.

## Supplementary Information

Below is the link to the electronic supplementary material.

Supplementary Material 1

## Data Availability

The datasets generated in this study are available from the corresponding author upon reasonable request.
