# Supplementary material for: Effectiveness of radiotherapy or chemoradiotherapy in older patients with advanced-stage, non-surgically treated head and neck cancer: A machine learning analysis
Source: Med Oncol. 2026 Oct 8;43(11):315. doi: 10.1007/s12032-026-03420-5 (PMC13649891; doi:10.1007/s12032-026-03420-5)
Supplement: Supplementary file 1 [file 12032_2026_3420_MOESM1_ESM.docx]

**Supplementary materials.**

The overall ML workflow is presented in **Supplementary Figure 1**.

**Supplementary Figure I:** A typical machine learning training process.

**Supplementary Figure II.** Standardized Mean Difference before and after propensity score matching for patients with HNSCC.

The detail description of the effect of CRT over RT has been presented in sub-sections 3.2 and 3.3 in the main manuscript.

**Supplementary Figures III-VI. Cox proportional hazards regression and Kaplan-Meier (KM) for Oropharyngeal squamous cell carcinoma (OPSCC).**

In terms of the multivariable Cox proportional hazards regression, M stage was the strongest adverse prognostic factor, demonstrating the largest increase in the hazard of disease-specific death. Increasing overall stage was also associated with poorer DSS, while advancing age conferred a modest but significant increase in mortality risk (**Supplementary Figure III**). In contrast, treatment with concurrent chemoradiotherapy (CRT) was associated with a reduced hazard of disease-specific death compared with radiotherapy (RT) alone, indicating a favorable survival benefit after adjustment for other covariates. Tumor grade also demonstrated a protective association with DSS, whereas N stage, sex, and marital status exhibited comparatively weaker effects. The magnitude and direction of the adjusted hazard ratios (HR) suggest that disease burden factors, particularly M stage and overall stage, exerted the greatest influence on survival outcomes for patients with OPSCC. CRT remained independently associated with improved DSS, supporting its role as a beneficial treatment strategy in patients with OPSCC. While some variables showed trends toward prognostic significance, their confidence intervals overlapped the null value, indicating limited independent effects after accounting for the influence of other clinical and demographic characteristics.

**Supplementary Figure III.** Forest plot of the multivariable Cox proportional hazards model demonstrating the association between clinicopathological variables and DSS for OPSCC.

Therefore, for OPSCC, M stage and overall cancer stage are the principal determinants of DSS, whereas CRT is independently associated with a clinically meaningful reduction in disease-specific mortality. In terms of the hazard ratios, increasing age was independently associated with poorer DSS (HR 1.03, 95% CI 1.00-1.05, *p*=0.03), indicating an approximately 3% increase in the hazard of disease-specific death for each additional year of age. M stage was the strongest adverse prognostic factor, with patients presenting with metastatic disease experiencing a more than twofold increased risk of disease-specific mortality compared with those without metastases (HR 2.63, 95% CI 1.24-5.57, *p*=0.01). Tumor grade was significantly associated with DSS (HR 0.73, 95% CI 0.54-0.99, *p*=0.04), suggesting that grade remained an independent prognostic factor after adjustment for other clinicopathological variables. Treatment modality demonstrated a clinically meaningful association with survival. Patients treated with CRT exhibited a 30% reduction in the hazard of disease-specific death compared with those receiving radiotherapy (RT) alone (HR 0.70, 95% CI 0.48-1.02, *p*=0.06) (**Supplementary Figure IV**).

**Supplementary Figure IV.** The hazard ratio (HR) and p-values for DSS in patients with OPSCC.

Although this association did not reach conventional statistical significance, it demonstrated a strong trend toward improved survival with CRT. Overall stage similarly showed a trend toward worse DSS (HR 1.46, 95% CI 0.93-2.31, *p*=0.10), whereas sex, marital status, and nodal stage were not significantly associated with disease-specific survival in the fully adjusted model (*p*>0.05 for all comparisons).

**Supplementary Figure V.** The Kaplan-Meier (KM) curve showing CRT is better than RT for DSS in patients with OPSCC.

The KM curve presented in **Supplementary Figure V** showed that CRT demonstrated improved DSS than RT in patients with OPSCC

**Supplementary Figure VI.** The propensity score matching (PSM) curve showed that CRT is better than RT for DSS in patients with OPSCC.

In terms of PSM, patients with OPSCC who received CRT showed better survival outcomes than their counterparts that received only RT (**Supplementary Figure VI)**.

**Supplementary Figure VII – X. Cox proportional hazards regression and Kaplan-Meier (KM) for laryngeal squamous cell carcinoma (LSCC).**

In terms of the multivariable Cox proportional hazards regression analysis, M stage was the strongest independent predictor of DSS (**Supplementary Figure VII**). Patients with metastatic disease exhibited a markedly increased risk of disease-specific mortality compared with those without metastasis. Increasing age was also independently associated with poorer survival outcomes, with each additional year of age contributing to a modest increase in the hazard of death.

**Supplementary Figure VII.** Forest plot of the multivariable Cox proportional hazards model demonstrating the association between clinicopathological variables and DSS for OPSCC.

Furthermore, sex was significantly associated with DSS, indicating an independent influence on survival after adjustment for other clinicopathological factors. Importantly, treatment modality remained an independent prognostic factor. Patients treated CRT experienced a significantly lower hazard of disease-specific death compared with those receiving RT alone, supporting a survival benefit associated with CRT. In contrast, tumor grade, marital status, overall stage, and N stage showed weaker associations with DSS after multivariable adjustment, although advanced stage and nodal involvement demonstrated trends toward worse survival (**Supplementary Figure VII**). Collectively, these findings indicate that M stage and age are the principal adverse determinants of DSS, whereas CRT is independently associated with improved survival outcomes in this cohort. Similarly, the HR in the multivariable Cox proportional hazards regression analysis for patients with LSCC showed that increasing age and M stage were independently associated with worse DSS. Each one-year increase in age was associated with a 4% increase in the hazard of disease-specific death (HR 1.04, 95% CI 1.02-1.05, *p*<0.005). M stage emerged as the strongest adverse prognostic factor, with patients presenting with M1 disease experiencing a nearly fourfold higher risk of disease-specific mortality compared with those without metastases (HR 3.78, 95% CI 2.34-6.11, *p*<0.005). Sex was also significantly associated with DSS (HR 1.32, 95% CI 1.01-1.71, *p*=0.04), indicating a modest but independent effect on survival outcomes (**Supplementary Figure VIII**).

**Supplementary Figure VIII.** The HR and p-values for DSS in patients with LSCC.

As with OPSCC, treatment modality remained an independent predictor of DSS after adjustment for all clinicopathological covariates. Patients treated with concurrent CRT experienced a significantly lower risk of disease-specific death compared with those receiving RT alone (HR 0.74, 95% CI 0.58-0.94, *p*=0.01), corresponding to an approximately 26% reduction in the hazard of mortality. In contrast, tumor grade, marital status, overall stage, and N stage were not significantly associated with DSS in the fully adjusted model (*p*>0.05 for all comparisons), although N stage demonstrated a trend toward poorer survival (HR 1.17, 95% CI 0.99-1.39, *p*=0.07). As shown in **Supplementary Figure VIII**, these findings indicate that age, M stage, and treatment modality are the principal independent determinants of disease-specific survival, with CRT conferring a significant survival advantage over RT alone. A similar result is obtained on the effect of treatment for KM curve in patients with LSCC (**Supplementary Figure IX**).

**Supplementary Figure IX**. The KM curve showing CRT is better than RT for DSS in patients with LSCC.

In terms of PSM, patients with LSCC who received CRT showed better survival outcomes than their counterparts that received only RT (**Supplementary Figure X)**.

**Supplementary Figure X.** The PSM curve showed that CRT is better than RT for DSS in patients with LSCC.

**Supplementary Figures XI-XII.**

**The permutation feature importance (PFI) analysis for both OPSCC and LSCC.**

The PFI for OPSCC showed that the overall TNM stage is the most important predictive feature for disease-specific survival (DSS) (**Supplementary Figure III**).

**Supplementary Figure XI.** The PFI analysis for patients with OPSCC.

The PFI analysis showed that the age of patients at diagnosis is the most important predictive feature for DSS in patients with LSCC.

**Supplementary Figure XII.** The PFI for patients with LSCC.
